# The organization and evolution of the *Responder* satellite in species of the *Drosophila melanogaster* group: dynamic evolution of a target of meiotic drive

**DOI:** 10.1186/s12862-014-0233-9

**Published:** 2014-11-25

**Authors:** Amanda M Larracuente

**Affiliations:** Department of Biology, River campus, University of Rochester, 480 Hutchison Hall, Rochester, NY 14627 USA

**Keywords:** Satellite DNA, Segregation Distorter, *Responder*, *Drosophila*, Meiotic drive, Concerted evolution

## Abstract

**Background:**

Satellite DNA can make up a substantial fraction of eukaryotic genomes and has roles in genome structure and chromosome segregation. The rapid evolution of satellite DNA can contribute to genomic instability and genetic incompatibilities between species. Despite its ubiquity and its contribution to genome evolution, we currently know little about the dynamics of satellite DNA evolution. The *Responder* (*Rsp*) satellite DNA family is found in the pericentric heterochromatin of chromosome *2* of *Drosophila melanogaster. Rsp* is well-known for being the target of *Segregation Distorter* (*SD*)— an autosomal meiotic drive system in *D. melanogaster*. I present an evolutionary genetic analysis of the *Rsp* family of repeats in *D. melanogaster* and its closely-related species in the *melanogaster* group (*D. simulans, D. sechellia, D. mauritiana, D. erecta,* and *D. yakuba*) using a combination of available BAC sequences, whole genome shotgun Sanger reads, Illumina short read deep sequencing, and fluorescence in situ hybridization.

**Results:**

I show that *Rsp* repeats have euchromatic locations throughout the *D. melanogaster* genome, that *Rsp* arrays show evidence for concerted evolution, and that *Rsp* repeats exist outside of *D. melanogaster,* in the *melanogaster* group. The repeats in these species are considerably diverged at the sequence level compared to *D. melanogaster,* and have a strikingly different genomic distribution, even between closely-related sister taxa.

**Conclusions:**

The genomic organization of the *Rsp* repeat in the *D. melanogaster* genome is complex—it exists of large blocks of tandem repeats in the heterochromatin and small blocks of tandem repeats in the euchromatin. My discovery of heterochromatic *Rsp-like* sequences outside of *D. melanogaster* suggests that *SD* evolved after its target satellite and that the evolution of the *Rsp* satellite family is highly dynamic over a short evolutionary time scale (<240,000 years).

**Electronic supplementary material:**

The online version of this article (doi:10.1186/s12862-014-0233-9) contains supplementary material, which is available to authorized users.

## Background

Genomes are frequently in conflict with selfish genetic elements that propagate in genomes or populations despite the harm that they cause to the host [1-3]. Genetic elements can range in their degree of selfishness from the expansion of blocks of tandemly repeated satellite DNAs [4] typically found near centromeres and telomeres [5], to the invasive properties of transposable elements or the ultra-selfish behavior of meiotic drivers. Meiotic drivers spread in populations by gaining a transmission advantage through gametogenesis [6]. *Segregation Distorter* (*SD)* is an autosomal male meiotic drive system in *Drosophila melanogaster* that has biased transmission— while heterozygous females transmit *SD* fairly to half of their progeny, heterozygous males transmit *SD* to nearly all of their progeny [7]. *SD* targets *Responder* (*Rsp*), a satellite DNA in the pericentric heterochromatin of *2R* [8,9]*.* The sensitivity of the *Rsp* locus to segregation distortion correlates positively with the number of repeats on *2R*—*SD* targets wild-type chromosomes with many *Rsp* repeats [8,9] resulting in wild-type sperm dysfunction through a currently unknown mechanism (reviewed in [10]).

The structure of the *Rsp* locus is complex: the canonical form of the repeat is a dimer of two related 120-bp repeats referred to as “Left” and “Right” *Rsp* (84% identical), but the tandemly arrayed canonical repeats are interspersed with more divergent variants of *Rsp* [11]. At least two additional locations of *Rsp* repeats exist outside of the *SD* target in *2R* pericentric heterochromatin: a cluster of repeats occurs on *3L* in cytological band 80C [12], and a single fragment of a *Rsp* repeat occurs on *2R* at cytological band 60A [11].

The evolutionary dynamics of the *Rsp* satellite are currently unknown. *SD* is a selfish genetic system specific to *D. melanogaster.* While the divergence between the Right and Left *Rsp* repeats suggests that the repeat is old, it has never been found outside of *D. melanogaster—*implying that the repeat arose in an ancestor of the *melanogaster* group and was subsequently lost outside of *D. melanogaster,* or it is rapidly evolving in *D. melanogaster*. These inferences resulted from studies based on DNA-DNA hybridization or poorly assembled and fairly low coverage genomes, however, and currently available genomic resources could provide new insights into the evolutionary history of *Rsp* repeat family evolution.

We know little about the evolutionary dynamics of satellite DNAs in general. Many repetitive DNAs undergo concerted evolution [13-15], whereby unequal recombination and/or gene conversion events cause repeats within a species to be more similar to each other than to their homologous repeats between species [15-20]. While concerted evolution is documented among various repetitive DNA sequences, the effect of intragenomic conflict on any particular family of repeats is understudied. While some satellite DNAs are expected to be selfish themselves, the *Rsp* repeats of *D. melanogaster* are instead (or perhaps in addition), the target of a selfish meiotic driver, making these especially interesting repeats to study. An evolutionary genetic analysis of the *Rsp* family of repeats could reveal important details about the evolutionary history of *SD*, as well as the dynamics of satellite DNAs and the effect of intragenomic conflict on genome evolution.

In this paper, I present an evolutionary genomic analysis of the *Rsp* satellite in *D. melanogaster* and the three closely related species of the *simulans* clade (*D. simulans, D. sechellia* and *D. mauritiana*). I combine traditional Sanger sequencing data (from BACs and Whole Genome Shotgun assemblies), Next Generation Sequencing (NGS) data (from genomic Illumina reads), and in situ hybridization to study patterns of *Rsp* satellite evolution on a short evolutionary time scale*.* I show that *Rsp* repeats have euchromatic locations throughout the *D. melanogaster* genome, that *Rsp* arrays show evidence for concerted evolution, and that *Rsp* repeats exist outside of *D. melanogaster,* in species of the *melanogaster* group*.* My analyses suggest that *Rsp* repeat family evolution is highly dynamic, and are consistent with the rapid evolution of *Rsp* in *D. melanogaster,* where it is a target of meiotic drive.

## Results and discussion

### Rsp in D. melanogaster

**Figure 1:**
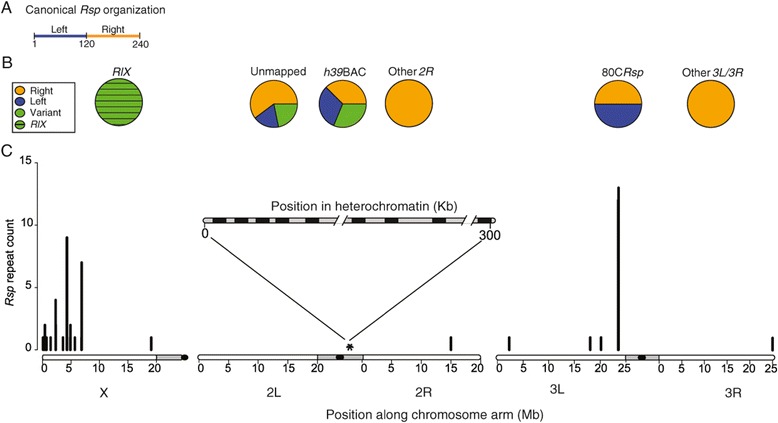
***Rsp*** **organization in the** ***D. melanogaster*** **genome. A.)** Organization of canonical *Rsp* repeats as a dimer of “Right” and “Left” repeats. **B.)** Pie charts showing the relative abundances of Right, Left *Rsp* and their variants, and *RlX* repeats, across the *D. melanogaster* genome. *h39* is a BAC that maps to the heterochromatic cytological band *h39*—the target of *SD. 80C Rsp* corresponds to repeats mapping to cytological band *80C* on chromosome *3L*. **C.)** *Rsp* repeat counts across the euchromatic *D. melanogaster* genome. Plotted are *Rsp* repeat counts along each chromosome arm in Mb of the euchromatic genome assembly. Shaded in grey are pericentric heterochromatin regions and black circles correspond to centromeres. A schematic of an assembly of unmapped BACs appears above the chromosome plots showing that blocks of *Rsp* repeats (in black) occur in clusters and are interspersed in the heterochromatin.

I used BLAST to identify *Rsp* repeats in the WGS assembly of *D. melanogaster* and found hits on nearly every chromosome arm (Figure 1). As expected, large blocks of canonical *Rsp* repeats—defined as the sequences similar to those correlated with the sensitivity to segregation distortion in Wu et al. [9]—occur on chromosome *2R* (these repeats in versions of the *D. melanogaster* genome prior to v6.01 were found in ArmU and ArmU extra scaffolds). These repeats correspond to the large block of satellite in the pericentric heterochromatin on chromosome *2R.* Consistent with previous findings [12], at least one large block of canonical *Rsp* repeats occurs on chromosome *3L.* However, I found several small blocks of *Rsp-*like repeats in the euchromatic regions of X, 2R, 3L and 3R (Figure 1B and C). Interestingly, the largest block of euchromatic repeats is found on chromosome *3L,* in an intron of the gene *Argonaute 3* (*Ago3*). The *Ago3* repeats are canonical *Rsp* repeats (Figure 1B). The euchromatic *Rsp* repeats consist of between 1 and 12 repeats which, because the assembly of repetitive sequences tends to collapse repeats with nearly-identical sequences, may be considered estimates of the minimum repeat number. The second largest euchromatic *Rsp* blocks occur on the X chromosome. The *Rsp-*like repeats found on the X chromosome are not canonical *Rsp* repeats, but divergent copies of a *Rsp* family repeat (hereon referred to as *RlX* for *Rsp-like on X*; *P-*value from permuted alignments with *D. melanogaster* canonical *Rsp* <10^−4^; Figure 1B)*.* There are three clusters of interspersed *RlX* repeats in cytological band 4C on the X chromosome (occurring within a 150 kb interval). The three clusters span 715 bp, 120 bp and 1430 bp, respectively, with the largest cluster occurring within 1 kb of the gene *CG12688*. I compared all individual WGS reads matching canonical *Rsp* to estimate genome-wide variability in *Rsp* repeat sequence. Overall, individual reads matching the Left and Right canonical *Rsp* sequences from across the genome shared 85.8% and 87.6% identity, indicating that there is considerable variability in canonical *Rsp* repeats in the genome.

Because during genome assembly, reads from identical or nearly identical sequences may be collapsed into a single sequence or mapped to the wrong location, repetitive regions of the genome are often misassembled [21]. This makes it difficult to reliably compare repeats in different regions of the WGS assembly. To compare repeats between regions of the genome, I instead used BLAST to identify *Rsp* repeats in sequenced BACs mapping to known genomic locations. I obtained hits on BACs that map to euchromatic and heterochromatic locations in the genome, including the *2R* heterochromatic division called *h39*, the target of *SD* [8]. The BAC categorized as *h39* (AC246306.1) contains clusters of *Bari-1* repeats that define the distal boundary of the *Rsp* locus at *h39* [22], (Additional file 1: Table S1). Within arrays (defined here as tandem blocks of *Rsp* repeats on non-overlapping BACs), I categorized repeats into the canonical Left repeats, canonical Right repeats, and variants of the canonical *Rsp* repeats. Among BACs that are unmapped and likely correspond to pericentric heterochromatin, there is less variability within Left (89.5 percent identity, 95% C.I. 79.5-100: Table 1) and Right (90.4 percent identity, 95% C.I. 82.3-100; Table 1) than between Left and Right repeats (82.4 percent identity, 95% C.I. 76.0-89.8; Table 1). The repeats that map to *h39* (the target of *SD*), appear more similar (i.e. fewer differences among repeats within the array) than the unmapped reads (Table 1). The repeats in the *3L* cluster are the most similar of the main blocks of *Rsp* repeats (Table 1). The *RlX* repeats are 52.5-57.7 percent identical (%ID) to the consensus canonical *Rsp* sequences over their entire length but well conserved over bases 57–110 in the consensus canonical *Rsp* sequences (*RlX* vs. Left *Rsp* is 76.7-79.1%ID; *RlX* vs. Right *Rsp* is 79.1-86%ID)*.* Moschetti et al. [12] posited that the original canonical *Rsp* repeats were identified from a clone that actually maps to *3L* instead of *h39.* Consistent with this idea, *Rsp* repeats found at 80C on chromosome *3L* are primarily the canonical *Rsp* sequences, whereas repeats found at *h39,* and unmapped BACs are a mix of canonical *Rsp* and their variants, where variants are defined as repeats that fall between 79.5%-90% identical to right or left canonical *Rsp* repeats (Figure 1B).

**Table 1:** **Percent identity within and between canonical** ***Rsp*** **repeat types, for BACs from unmapped regions,** ***h39*** **(target of** ***SD*** **)** ***,*** **and** ***3L***

**BAC location**	**Within-repeat type %ID**		**Between-repeat type %ID**
	**Left**	**Right**	**Left-right**
Unmapped	89.5 (79.5-100)	90.4 (82.3-100)	82.4 (76.0-89.8)
*h39*	99.6 (99.1-100)	92.1 (84.1-100)	81.5 (80.6-82.3)
*3L*	96.5 (93.0-100)	90.1 (94.5-100)	79.4 (76.2-91.9)

Canonical repeats have the little variation on *3L* and at *h39*, but are highly variable on the unmapped BAC. Variability between Left and Right canonical repeats does not differ between regions.

**Figure 2:**
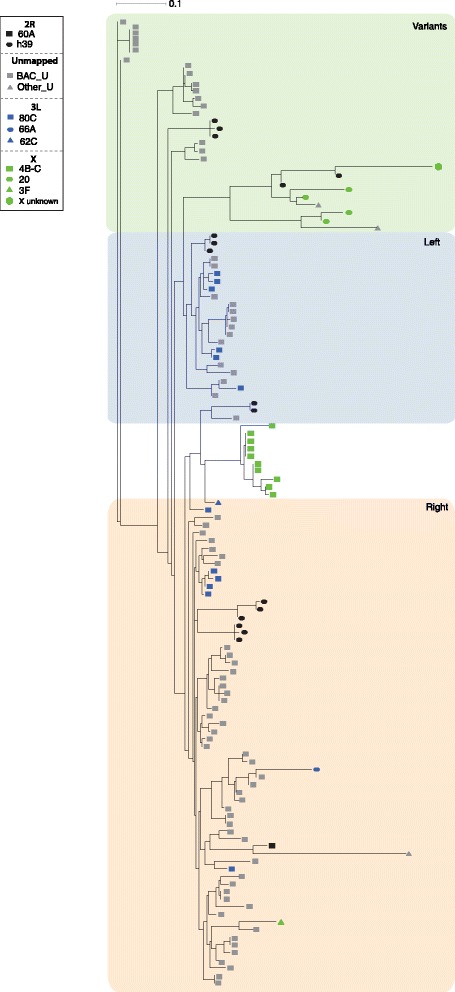
***D. melanogaster Rsp*** **repeat clusters across the genome.** Plotted is a neighbor-joining tree of *Rsp* repeats on BACs across the genome. BACs are color-coded according to location.

A neighbor-joining tree constructed from *Rsp* repeats found in clusters on different BACs confirms that repeats within an array tend to be more similar to each other than repeats outside of an array (Figure 2), a pattern consistent with a history of concerted evolution. Surprisingly, for some repeat clusters, it appears that there is even exchange between arrays of repeats in different genomic locations and perhaps even different chromosome arms (between unmapped BACs whose likely location is chromosome *2R* and *3L* BACs; Figure 2). It is however possible that the unmapped BACs map to pericentric heterochromatin on *3L* instead of *2R,* however if this were the case, this result still demonstrates exchange between distinct clusters of repeats (no portion of *Ago3* occurs on this unmapped BAC). Unfortunately, I was unable to conclusively determine the genomic location of the unmapped BACs [23], (Additional file 1: Figure S1). The *Rsp* repeats found on BACs mapping to *2R* (at *h39*) and *3L* are very similar [23] (Additional file 1: Table S2), consistent with previous observations [24]. While this may be evidence for rare interchromosomal exchange between *2R* and *3L,* such exchange has not been documented for other repeat families in *Drosophila*. Alternatively, selective sweeps involving the *2R* pericentric heterochromatin could also cause repeats on different chromosomes to be more closely related to each other than nearby repeats, as the different chromosomes would have the same recent common ancestor [24].

### Rsp family repeats in simulans clade species

Although the *Rsp* satellite has not been described outside of *D. melanogaster* [9,24,25], I found repeats similar to *Rsp* in each species of the *simulans* clade, and in *D. erecta* and *D. yakuba* (hereon referred to as *Rsp-like*; [23], Additional file 1: Table S3). I used BLAST to identify sequences related to the *D. melanogaster Rsp* repeat in the WGS assemblies of *D. sechellia, D. simulans, D. erecta* and *D. yakuba* (*P-*values from permuted alignments with *D. melanogaster* canonical *Rsp* are <10^−4^ for each species)*.* The repeat unit of *Rsp-like* is larger than canonical *Rsp: Rsp-like* is ~160 bp in the *simulans* clade and 173 bp in *D. erecta,* whereas canonical *Rsp* in *D. melanogaster* is ~120 bp. Throughout the paper, only the ~120 bp homologous to canonical *Rsp* is analyzed. Interestingly, *D. erecta Rsp-like* repeats appear to have a dimeric structure analogous to the Left and Right repeats of *D. melanogaster.* I refer to the *D. erecta* repeats as *Rsp-like-1* and *Rsp-like-2* (there is 9.5% divergence between the pairs) to avoid confusion with the Left and Right repeats of *D. melanogaster* because the dimeric structures appear independently derived.

Because repetitive sequences are often underrepresented and misassembled in traditional WGS assemblies [21,26,27], I also queried the reads of Illumina NGS datasets in *D. melanogaster* and species of the *simulans* clade—*D. simulans, D. sechellia* and *D. mauritiana—*for *Rsp* sequences. Using the consensus of *Rsp-like* sequences as references, I identified *Rsp* and *Rsp-like* repeats among the NGS reads of *D. melanogaster, D. sechellia, D. simulans,* and *D. mauritiana* using Bowtie2 (KP016744-KP016746)*.* I collected all unique *Rsp* sequences (individual repeat units) by constructing *de novo* assemblies of all canonical *Rsp* and *Rsp-like* reads for each species (see Methods; Table 2). Species vary widely in their number of unique repeats: *D. sechellia,* in particular, has an order of magnitude more unique repeats than the other species (Table 2).

**Table 2:** **Number of unique** ***Rsp*** **or** ***Rsp-like*** **repeats in** ***D. melanogaster*** **or the** ***simulans*** **clade, respectively**

**Species**	**Unique repeats** ^**a**^
*D. melanogaster*	316
*D. simulans*	164
*D. sechellia*	1738
*D. mauritiana*	32

^a^The number of unique *Rsp* or *Rsp-like* repeats extracted from the *de novo* assemblies of *NGS* reads (see Methods).

**Figure 3:**
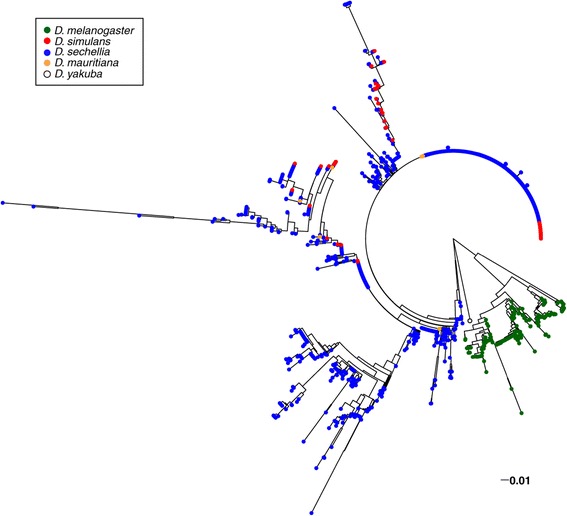
**Neighbor joining tree of unique** ***Rsp*** **sequences in** ***D. melanogaster*** **and** ***Rsp-like*** **sequences in species of the** ***simulans*** **clade with** ***D. yakuba*** **as the outgroup suggests that** ***Rsp*** **evolves rapidly in** ***D. melanogaster*** **.** These unique sequences were compiled from the NGS reads in each species. The *Rsp-like* sequences of *D.simulans* (red), *D. sechellia* (blue) and *D. mauritiana* (orange) are interleaved throughout the tree whereas the canonical Rsp in *D. melanogaster* (green) forms its own clade.

Because several indels differentiate repeats within and between species, I constructed a neighbor joining tree based on distance using a model that considers adjacent indels as a 5th nucleotide state. This tree was constructed for unique *Rsp* and *Rsp-like* sequences (excluding *RlX* repeats and partial repeat units) in *D. melanogaster* and species of the *simulans* clade with *D. yakuba* as the outgroup [23]. The tree topology reveals that *Rsp-like* sequences in *D. simulans, D. sechellia and D. mauritiana* are very similar (Figure 3)*.* The divergence between the canonical *D. melanogaster* Left and Right *Rsp* (79-82% ID) implies that the repeat family originated before the speciation of *melanogaster* group species, assuming a molecular clock. However, the *Rsp* repeats in *D. melanogaster* form a monophyletic group, whereas the *Rsp-like* repeats of the *simulans* clade species are intercalated throughout the tree (Figure 3). I also inferred the best maximum likelihood tree for all unique *Rsp* and *Rsp-like* sequences and found the same pattern [23], (Additional file 1: Figure S2). The canonical *D. melanogaster Rsp* and *simulans* clade species *Rsp-like* repeats are highly similar in the first 28 bp and the last 66 bp, but difficult to align in the middle. I am therefore uncertain of the length of the internal branch leading to the *D. melanogaster* repeats (Figure 3). Taken together, the excess divergence and monophyly of *Rsp* repeats on the *D. melanogaster* branch implies that there has been accelerated evolution in this lineage, where it is the target of *SD.*

### Relationship between heterochromatic and euchromatic repeats

**Figure 4:**
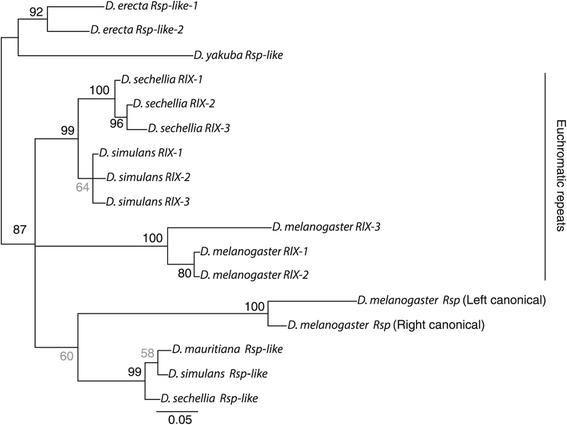
***Rsp*** **family satellite evolution.** Bayesian tree showing the relationship between three orthologous euchromatic *RlX* repeats in *D. melanogaster, D. sechellia* and *D. simulans,* and the heterochromatic *Rsp-like* sequences of the *simulans* clade species, *D. erecta,* and *D. yakuba*, and the canonical (Left and Right) *Rsp* repeats of *D. melanogaster.* The *Rsp-like* repeats of the *simulans* clade species represent the consensus of all unique *Rsp-like* repeats assembled from the NGS reads (see Methods) and the *Rsp-like* repeats of *D. erecta* and *D. yakuba* represents the consensus of all *Rsp-like* repeats in the WGS assembly. *D. erecta* has two related *Rsp-like* repeats: *Rsp-like-1* and *Rsp-like-*2. Posterior probability as a percent is indicated at the nodes, with values <80% shaded in grey. The tree was rooted post-hoc at the ancestor of *D. yakuba* and *D. erecta.*

In addition to the heterochromatic *Rsp-like* repeats, the WGS assemblies of *D. sechellia* and *D. simulans* include the euchromatic *RlX* repeats. To examine the relationship between the heterochromatic *Rsp* and *Rsp-like* repeats and the euchromatic *RlX* in *D. melanogaster, D. simulans, D. sechellia, D. mauritiana, D. erecta* and *D. yakuba,* I constructed phylogenies using MrBayes. To test for concerted evolution of the euchromatic *RlX* repeats, I selected three *RlX* repeats occurring in orthologous positions upstream of the X-linked gene CG12688 for *D. melanogaster, D. sechellia* and *D. simulans.* The tree suggests that the *D melanogaster* canonical *Rsp* repeats are indeed monophyletic and evolving rapidly. The tree topology indicates that the *Rsp-like* repeats of the *simulans* clade groups with canonical *Rsp* repeats of *D. melanogaster*, but with low posterior probability (60%; Figure 4). Comparing overall percent identity, the *RlX* repeats are more similar to the *Rsp-like* repeats of the *simulans* clade than canonical *D. melanogaster Rsp* repeats: whereas *RlX* to *Rsp-like* percent identify varies between 75.6-80.7%, *RlX* to *Rsp* (canonical) only varies from 55.6-61.5% [23], again suggesting that canonical *Rsp* repeats evolve rapidly in *D. melanogaster*. Although it is possible that canonical *Rsp* is an old repeat that was lost in the *simulans* clade, the tree topology suggests that this is not the case (Figure 4). The polytomy between the *simulans* clade *RlX* repeats, the *D. melanogaster RlX* repeats and the heterochromatic *Rsp* and *Rsp-like* repeats of *D. melanogaster* and the *simulans* clade (Figure 4) may be influenced by gene conversion events between *RlX* repeats and canonical *Rsp* repeats in *D. melanogaster* [23]*.* A comparison of the three tandem *RlX* repeats in orthologous positions on the X chromosome (in cytological band 4C upstream of *CG12688*) revealed a pattern of concerted evolution: repeats within a species are more similar than repeats at the orthologous positions between species (Figure 4).

### Dynamic genomic distribution of Rsp-like repeats

**Figure 5:**
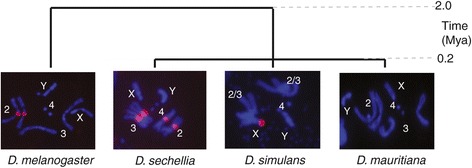
**FISH showing** ***Rsp*** **satellite blocks in** ***D. melanogaster*** **and species of the** ***simulans*** **clade.** *Rsp* and *Rsp-like* repeats are evolutionarily dynamic at the chromosome level.

The WGS assembly and NGS reads cannot give a complete picture of the satellite DNA distribution in these genomes because of assembly difficulties in heterochromatin. To determine the large-scale organization of the *Rsp* repeats in these species, I used FISH on mitotic chromosomes from larval neuroblasts using probes specific to *D. melanogaster Rsp* (for *D. melanogaster* FISH) or *D. sechellia Rsp* (for *D. sechellia, D. simulans* and *D. mauritiana* FISH). Small euchromatic satellite repeat islands are not detectable at this resolution, instead the FISH reveals the locations of large blocks of tandem satellite repeats. I discovered that the amount and distribution of *Rsp-like* repeats varies between the species. *Rsp-like* repeats exist as large blocks of satellite DNA in *D. sechellia* and *D. simulans* but not *D. mauritiana* (they are not detectable using FISH on mitotic figures; these repeats are also not detectable in *D. yakuba,* data not shown)*.* The *D. melanogaster* probe does not cross hybridize with *simulans* clade species and the *D. sechellia* probe does not cross hybridize with *D. melanogaster.* Furthermore, the genomic location of *Rsp*-*like* satellite blocks has changed dramatically between species. Whereas in *D. melanogaster,* the *Rsp* satellite is located in the pericentric region of *2R,* in *D. sechellia* the *Rsp-like* satellite is in the pericentric region of *2R, 3R* and *3L*, and in *D. simulans,* the *Rsp-like* satellite occurs only at the base of the X chromosome (Figure 5). The chromosome arms of these species are homologous and have extremely high degrees of synteny [28]—they appear to mostly differ at a gross scale in their distribution of satellite repeats.

## Conclusions

We currently know little about the evolution of satDNA, despite that it can make up a significant fraction of eukaryotic genomes [29]: >50% of the genome in kangaroo rats [30] and tenebrionid beetles [31]. Because of its ability to spread in genomes without offering any benefit to the host, satDNA has been considered selfish, junk DNA [1-3]. However, subsequent work in evolutionary, molecular, cellular, and cancer biology has converged on the idea that the heterochromatic fraction of the genome, including satDNAs, has important functional consequences [32-46]. While some blocks of satellite DNA themselves may exhibit meiotic drive, or biased transmission through gametogenesis [6] in females [47,48], the topic of this study—the *Responder* (*Rsp*) satellite—is a instead the target of meiotic drive in male *D. melanogaster* [7]. My discovery of highly dynamic evolution in the *Rsp* satellite family offers a model system to study the evolutionary dynamics of satDNA and the genetic conflict surrounding this enigmatic compartment of the genome.

Most of what we know about satellite DNA dynamics is at the resolution of large blocks of satellite DNA on chromosome arms. Assembly issues with repetitive DNA have stymied our understanding of satellite DNA evolutionary dynamics at the genomic level [21]. Using information from the physical map (e.g. BACs [27,49]) and deep Illumina sequencing in combination with FISH offers a higher resolution image of satellite DNA evolution within and between species. My analysis of the *Rsp* satellite DNA family demonstrates that *Rsp*: *1)* exists outside of the large block of pericentromeric satellite in locations across the *D. melanogaster* genome; *2)* exists in species of the *melanogaster* group; *3)* shows evidence for concerted evolution; and *4)* is evolutionary dynamic in its abundance and genomic distribution over short evolutionary time scales.

### Euchromatic satellite repeat islands

The *Rsp* satellite is a particularly interesting satellite DNA because it is the target of the *SD* meiotic drive system in *D. melanogaster*. Moschetti et al. [12] showed using FISH on polytene chromosomes that a small block of *Rsp* exists on chromosome *3L,* outside the region associated with *SD* (at heterochromatic division *h39* on *2R*)*.* I show here that in addition to the block of repeats on *3L, Rsp* family repeats exist in “euchromatic satellite repeat islands” in locations across the *D. melanogaster* genome, most notably on *3L* and the X chromosome (*RlX* repeats). Variable tandem repeats in several taxa have been considered as possible sources of gene regulatory variation (reviewed in [50]). It is possible that these *Rsp* repeat islands have some regulatory role in the expression of nearby genes or local chromatin condensation: in some cases they occur in or near genes in *D. melanogaster*. Similar to the genomic distribution of *Rsp,* short, euchromatic blocks of up to 5 tandem repeats were recently reported clustering in or near genes for satellites of the 1.688 family [14] in *D. melanogaster*.

The largest euchromatic *Rsp* islands are on *3L* and the X chromosome. The X chromosome has a special role in the *SD* system: escapers from *SD*-mediated meiotic drive have biased sex ratio [51], and X-linked suppressors of *SD* segregate at high frequencies in natural populations [52,53]. Some X chromosomes therefore seem to offer a protective effect against *SD*. Factors that suppress *SD* map to at least three regions of the X chromosome [54], and some of these intervals contain *RlX* repeats. It will be interesting to determine if the *RlX* repeats are involved in suppression of the *SD* system. One recently proposed hypothesis for the molecular mechanism of segregation distortion suggests that repeat-associated small interfering RNAs (rasiRNAs) corresponding to *Rsp* are necessary for proper packaging of the satellite during spermiogenesis, and that *SD* interferes with the production or localization of these rasiRNAs [10,55,56]. Many satellite DNAs in *D. melanogaster* [37] and other taxa [34,57,58] are transcribed and processed into small RNAs, including *Rsp* ([59]; Larracuente, unpublished). One possibility is that these euchromatic *Rsp* satellite repeat islands correspond to *Rsp* rasiRNA-producing clusters.

### Dynamic satellite DNA evolution on small time scales

*SD* is a *melanogaster-*specific drive system [60,61], but the history of the *Rsp* satellite has been unclear. While divergence between the Left and Right canonical *Rsp* repeats suggests that the repeat is old, the repeats had not been reported outside of *D. melanogaster* previously [9], although some studies indicated that divergent copies of the repeat may exist in *D. simulans* [24,25]. My results demonstrate conclusively that *Rsp*-*like* sequences exist in abundance in the closely-related species of the *simulans* clade, adding important insight into the evolution of the *SD* system—*SD* arose in *D. melanogaster* in a background that already had *Rsp* repeats to target. The *D. melanogaster* canonical *Rsp* is highly divergent compared to the *Rsp-like* repeats of the *simulans* clade species (e.g. 56-63% ID) and monophyletic, suggesting that *Rsp* has accelerated evolution in *D. melanogaster.* What might drive the rapid evolution of this repeat family in *D. melanogaster*? One possibility is that the mutation rate is unusually high for *D. melanogaster Rsp* compared to the *Rsp-like* repeats in the *simulans* clade species. An alternative explanation is that in *D. melanogaster,* segregation distortion against the canonical *Rsp* repeats by *SD* creates selection pressure to diverge from the target sequence. At present, we do not know the molecular mechanism of distortion, or what feature of the *Rsp* locus makes it a target (i.e. if it is sequence-specific). Efforts to compare the detailed evolutionary history of *Rsp* family repeats in the *melanogaster* group are underway.

Comparing *Rsp* repeat sequences within and between species revealed that *Rsp* and *Rsp-like* sequences show at least two lines of evidence for concerted evolution: *1)* repeats within the *D. melanogaster* genome are more similar within a genomic cluster than between genomic clusters; and *2) RlX* repeats between *D. melanogaster* and species of the *simulans* clade are most closely related to nearby repeats within each species than to repeats at orthologous positions between species.

At least in part due to the mutational properties of repetitive DNA [62], the turnover in satellite DNAs between closely-related species can be extreme [5,63] and in some cases may contribute to genetic incompatibilities between species [5,38]. On a genome-wide level, *Rsp* family evolution is highly dynamic. In the time since *D. sechellia, D. simulans* and *D. mauritiana* diverged—approximately 240 Kya [64]—the *Rsp-like* satellite has dramatically changed its genomic distribution. In *D. melanogaster,* large blocks of the *Rsp* satellite occur in the pericentric heterochromatin of *2R,* in *D. sechellia,* the *Rsp-like* satellite expanded and occurs in the pericentric heterochromatin of *2R, 3L* and *3R,* in *D. simulans,* the *Rsp-like* satellite only occurs at the base of the X chromosome and the *Rsp-like* satellite is undetectable at the chromosome level in *D. mauritiana* and *D. yakuba. Rsp* repeat copy number is highly polymorphic within *D. melanogaster* [65]*—*this polymorphism is directly related to *SD,* as large blocks of satellite confer sensitivity to segregation distortion [8,9]*.* The *Rsp-like* repeats in non-*melanogaster* species are presumably not targets of a meiotic drive system. It will be interesting to compare the population genetics of *Rsp* between species where it is and is not a target of meiotic drive.

## Methods

### Querying Whole Genome Shotgun (WGS), BAC assemblies, and reads

*D. melanogaster* reads were downloaded from the NCBI Trace Archive; BACs [27,49] and WGS contigs [66] were downloaded from Genbank. *Rsp* repeats were found in the *D. melanogaster* WGS genome assembly (version 6.01), BACs and NCBI Trace Archive using local basic local alignment search tool (BLAST) searches. Individual traces from four BACs (AC246323.1, AC246299.1, AC007548.10, and AC009843.9; [23], Additional file 1: Figure S1) were obtained from Sue Celniker and Kenneth Wan. An iterative BLAST search protocol was used to gather all sequences matching *Rsp.* Initial BLAST searches were performed using *Rsp* sequences deposited in Genbank as queries. To capture as much variation in *Rsp* sequence as possible, and to recover more divergent *Rsp* sequences, several BLAST iterations were performed in which subsequent BLAST searches used incrementally refined lists of the hits from previous BLAST searches as queries. This iterative process ended when no additional significant hits were obtained. BLAST hits with an *e-*value >0.1 and a length less than 30 bp were excluded from the analysis. BLAST alignments for hits with an *e*-value > .001 and length <50 were inspected by eye. Redundancies between sequences were removed using custom Perl scripts. Alignments of *Rsp* sequences were made using BWA-SW [67]. Pairwise percent identity between *Rsp* repeats was calculated in Geneious (version 6.1.7, created by Biomatters; http://www.geneious.com; [68] and the mean and 95% confidence intervals of pairwise percent identity were calculated in R. The same procedure was used to query the WGS assemblies of *D. sechellia* [28], *D. simulans* [69]*, D. erecta* [28], and *D. yakuba* [28]. To determine that *Rsp* and *Rsp-like* repeats are indeed related sequences, the nucleotides of each repeat sequence were randomly shuffled and percent identity was re-calculated for pairwise alignments using custom perl scripts. To create a distribution of percent identity based on nucleotide composition, 10,000 permutations were completed for each sequence and P-values were obtained using the empirical cumulative distribution function for each set of permuted alignments (in R).

### Querying Next Generation Sequencing (NGS) reads

Illumina GAIIX paired end reads from *D. melanogaster* (SRR060098; [70])*, D. simulans* (SRR520350; [69])*, D. sechellia* (SRR869587; [64]) and *D. mauritiana* (SRR483621; [64]) were downloaded from the NCBI’s SRA (http://www.ncbi.nlm.nih.gov/sra). NGS reads were trimmed of adapters and low quality bases using Trim Galore (version 0.2.8; Babraham Bioinformatics http://www.bioinformatics.babraham.ac.uk/projects/trim_galore/). Using Bowtie2 [71], the trimmed reads were mapped to consensus *Rsp* sequences (from the iterative BLAST procedure described above) that capture variation within and between species from WGS and BAC sequences. The alignments were analyzed using Samtools-0.1.18 [72]. To create a list of unique *Rsp* sequences for each species, *de novo* assemblies were constructed using the mapped reads extracted from SAM files created from Bowtie2. ABySS (version 1.3.6; [73] was used for the *de novo* assembly with the following parameters: *k* =64; *se*; m = 30; *l* =64. The unique contigs were parsed for individual *Rsp* sequences using BLAST to the original query *Rsp* and custom Perl scripts (e.g. a 400-bp contig was split into three individual *Rsp* repeats as determined by BLAST to a consensus *Rsp* sequence). Some assembled repeat units were less than the canonical repeat unit length (~120 bp). Because some of these shorter units represent true fragmented repeats and some are expected to be fragments of unique repeats that the *de novo* technique couldn’t complete (for reasons such as insufficient read depth or sequencing error), repeats with identical sequence but length variants were merged. Specifically, short, overlapping fragments with identical sequence and overhangs (non-overlapping sequence) of <50% of the total fragment length were merged into a single unique repeat. The list of unique *Rsp* repeats in each species was imported into Geneious (version 6.1.7 [68] to create and edit alignments.

### Trees

Neighbor-joining trees (Saitou and Nei 1987) were constructed using bionj with the ape package in R. Blocks of adjacent indels were included in the model using the setting “model = indelblock” and bootstrapping was done using the boot.phylo function in the ape package (B = 100). Bayesian inference of the phylogenetic relationships between the *RlX, Rsp-like* and canonical *Rsp* repeats was performed using MrBayes [74] using a GTR nucleotide substitution model with Gamma rate variation. The *Rsp-like* sequences of the *simulans* clade species were consensus sequences (GenBank accession numbers: KP016744, KP016745, KP016746) of the *de novo* contigs assembled from each species NGS reads. The *Rsp-like* repeats of *D. yakuba* and *D. erecta* are consensus sequences from the BLAST of WGS assemblies. The *RlX-1, RlX-2* and *RlX-3* are orthologs of three tandemly-repeated *RlX* sequences from cytological band 4C in the region ~1 kb upstream of *CG12688*.

Maximum likelihood inference of the best tree for the unique *Rsp* sequences from the NGS reads of *D. melanogaster, D. sechellia, D. simulans* and *D. mauritiana* was performed in RAxML v7.4.2 [75] using the CIPRES Gateway (http://www.phylo.org); [76]. Bootstrapping was performed using a GTR plus Gamma nucleotide substitution model (−m GTRGAMMA –× 1000). The maximum likelihood tree was drawn using the ape package in R [77]. The full tree including bootstrap confidence at the nodes is deposited in the Dryad Digital Repository (http://dx.doi.org/10.5061/dryad.3sh6d; [23]).

### Fluorescence in situ hybridization (FISH)

FISH to mitotic chromosomes in larval neuroblasts was performed as previously described [78,79]. Briefly, brains were dissected from 3rd instar larvae and fixed in 1.8% paraformaldehyde, 45% acetic acid. Fixed brains were denatured at 95**°**C and hybridized overnight at 30**°**C. Slides were washed in 4X SSCT three times, and 0.1X SSC three times before mounting in Vectashield with DAPI. The probe was a biotinylated, nick translated PCR product specific to *Rsp* repeats *D. melanogaster* (F-5’ GGAAAATCACCCATTTTGATCGC and R-5’ CCGAATTCAAGTACCAGAC for *D. melanogaster* FISH) or *Rsp-like* repeats in *D. sechellia* (F-5’ ACTGATTATCATCGCCTGGT and R-5’ TCCAGTTCGCCTGGTAGTTT; for *D. sechellia, D. simulans,* and *D. mauritiana* FISH).

## Availability of supporting data

The data sets supporting the results of this article are available in the Dryad repository, [doi:10.5061/dryad.3sh6d and http://dx.doi.org/10.5061/dryad.3sh6d] and GenBank (KP016744-KP016746).

## Additional file

Additional file 1: Table S1. BACs used in this paper. **Table S2.** Repeat comparison between BACs mapping to U and 3L (80C-D) and h39. Reported are pairwise percent identity (%ID) and 95% confidence intervals (95% C.I.). **Table S3.** Percent identity between consensus *Rsp, Rsp-like* and *RlX* repeats. **Figure S1.** Maximum likelihood tree showing relationship between *Rsp* and *Rsp-like* repeats the *melanogaster* group. **Figure S2.** FISH with digested, unmapped BAC maps to both *2R* and *3L/3R* in mitotic chromosomes from brains. **Figure S3.** Alignment of heterochromatic *Rsp* family repeats (canonical *Rsp* of *D. melanogaster* and *Rsp-like* of the *simulans* clade, *D. erecta* and *D. yakuba* repeats) and euchromatic *RlX* repeats in species of the *melanogaster* subgroup.

## Abbreviations

WGS: Whole genome shotgun
NGS: Next generation sequencing
*Rsp*: *Responder*
*Rsp-like*: *Responder-like*
*RlX*: *Responder-like on X*
*SD*: *Segregation distorter*
BAC: Bacterial artificial chromosome
FISH: Fluorescence in situ hybridization
