# Supplementary material for: The organization and evolution of the Responder satellite in species of the Drosophila melanogaster group: dynamic evolution of a target of meiotic drive
Source: BMC Evol Biol. 2014 Nov 25;14:233. doi: 10.1186/s12862-014-0233-9 (PMC4280042; doi:10.1186/s12862-014-0233-9)
Supplement: Additional file 1: Table S1. — BACs used in this paper. Table S2. Repeat comparison between BACs mapping to U and 3L (80C-D) and h39. Reported are pairwise percent identity (%ID) and 95% confidence intervals (95% C.I.). Table S3. Percent identity between consensus Rsp, Rsp-like and RlX repeats. Figure S1. Maximum likelihood tree showing relationship between Rsp and Rsp-like repeats the melanogaster group. Figure S2. FISH with digested, unmapped BAC maps to both 2R and 3L/3R in mitotic chromosomes from brains. Figure S3. Alignment of heterochromatic Rsp family repeats (canonical Rsp of D. melanogaster and Rsp-like of the simulans clade, D. erecta and D. yakuba repeats) and euchromatic RlX repeats in species of the melanogaster subgroup. [file 12862_2014_233_MOESM1_ESM.pdf]

**Accession number**	**BAC name**	**Genomic location**
AC023695.4	RP98-25I9	X
AC185536.1	BACR38K14	X (20B)
AC007548.10	BACR48M17	3L (80B1-D2)
AC009843.9	BACR01G11	3L (80B1-D2)
AC008214.4	BACR13P17	3R (97B-97C)
AC154045.1	BACR34K23	3L
CR942806.6	BACR-15A01	Unmapped
AC091225.2	RP98-34B24	3L
AC012165.6	BACR48P17	X (18F)
AC016368.3	BACR36A03	2R (42A-42B)
AC018482.3	BACR16N12	3R (81)
AC185538.1	BACR04D19	2R (42B)
AC246306.1	BACR32B23	*h39***
AC246323.1	CH221-04O17	Unmapped
AC246299.1	BACN05C06	Unmapped
AC009458.7	BACR30H12	3L (80B1-80D2)
AC023742.4	RP98-43C24	X (4B6-4C4)
AC108481.2	RP98-1O19	X (4B-4C)*
AC023725.5	RP98-9I10	X
AC105352.2	RP98-45O17	X (4A-B)
AC091204.3	RP98-27G13	3L (66A)
AC154044.2	BACR32E02	X (3F)
AC099018.1	BACR28M05	2R (60A)

Table S1. BACs used in this paper. *BAC RP98-1O19 is listed as mapping to 3L, however this actually maps to cytological bands 4B-4C on the X chromosome. **BACR32B23 is listed as unmapped, however the unique Bari-I repeats that define the distal boundary of the *Rsp* cluster at *h39* on chromosome *2R* are present on this BAC, therefore I assign this BAC to *h39*.

Repeat	%ID	95% C.I.
	**U *vs* 3L**	
**Left**	89.3	82.4-95.7
**Right**	89.9	83.5-95.1
	**3L *vs* h39**	
**Left**	89.8	88.5-91.5
**Right**	86.5	82.0-88.9
	**U *vs* h39**	
**Left**	90.2	87.5-93.0
**Right**	86.3	80.9-90.4
**V1**	80.5	76.8-84.6

Table S2. Repeat comparison between BACs mapping to U and 3L (80C-D) and h39. Reported are pairwise percent identity (%ID) and 95% confidence intervals (95% C.I.).

Table S3. Percent identity between consensus *Rsp, Rsp-like* and *RlX* repeats.

	Rsp_{Dmel} (L)	Rsp_{Dmel} (R)	$Rsp\text{-}like_{Dsec}$	$Rsp\text{-}like_{Dsim}$	$Rsp\text{-}like_{Dmau}$	$Rsp\text{-}like_{Dyak}$	$Rsp\text{-}like\text{-}1_{Dere}$	$Rsp\text{-}like\text{-}2_{Dere}$	RlX_{Dmel}	RlX_{Dsec}
Rsp_{Dmel} (L)										
Rsp_{Dmel} (R)	86.7									
$Rsp\text{-}like_{Dsec}$	55.6	60.7								
$Rsp\text{-}like_{Dsim}$	57.8	63.0	96.3							
$Rsp\text{-}like_{Dmau}$	57.8	63.0	96.3	98.5						
$Rsp\text{-}like_{Dyak}$	49.6	50.3	54.1	56.3	56.3					
$Rsp\text{-}like\text{-}1_{Dere}$	51.1	55.1	74.4	74.2	74.2	58.2				
$Rsp\text{-}like\text{-}2_{Dere}$	51.9	54.4	73.6	73.4	73.4	60.0	90.5			
RlX_{Dmel}	55.6	58.5	75.6	77.8	76.3	53.3	67.5	67.5		
RlX_{Dsec}	57.0	60.7	77.8	79.3	79.3	54.1	72.7	73.5	80.7	
RlX_{Dsim}	57.8	61.5	79.2	80.7	80.7	52.6	75.2	76.1	77.8	96.3

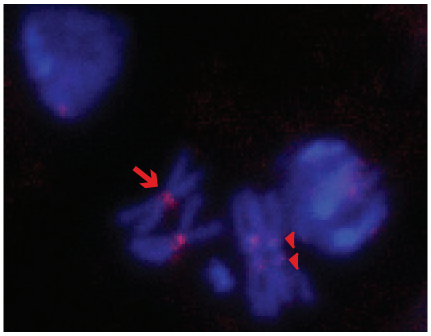

Figure S1. FISH with digested, unmapped BAC maps to both *2R* and *3L/3R* in mitotic chromosomes from brains. In an effort to remove most *Rsp* repeats from the unmapped BAC CH221-04O17 (AC246323.1), the BAC was digested with Xba1—a restriction site in canonical *Rsp*—and large bands were gel purified, nick translated and biotinylated (with BioNick labeling system) to make a probe. Because this BAC contains repetitive elements common to both *2R* and chromosome *3* pericentric heterochromatin (that presumably are not enriched for *Rsp* repeats), I was unable to map this BAC uniquely. Although note that the staining at *2R* is strong (red arrow) and the probe only weakly stains *3R* and *3L* pericentric heterochromatin (red arrowheads), this does not necessarily imply that the BAC originates from *2R*. Undigested *Rsp* repeats are unlikely to contribute to this pattern because a probe with only *Rsp* repeats does not produce staining on chromosome *3* (Figure 5)—there are too few repeats to detect.

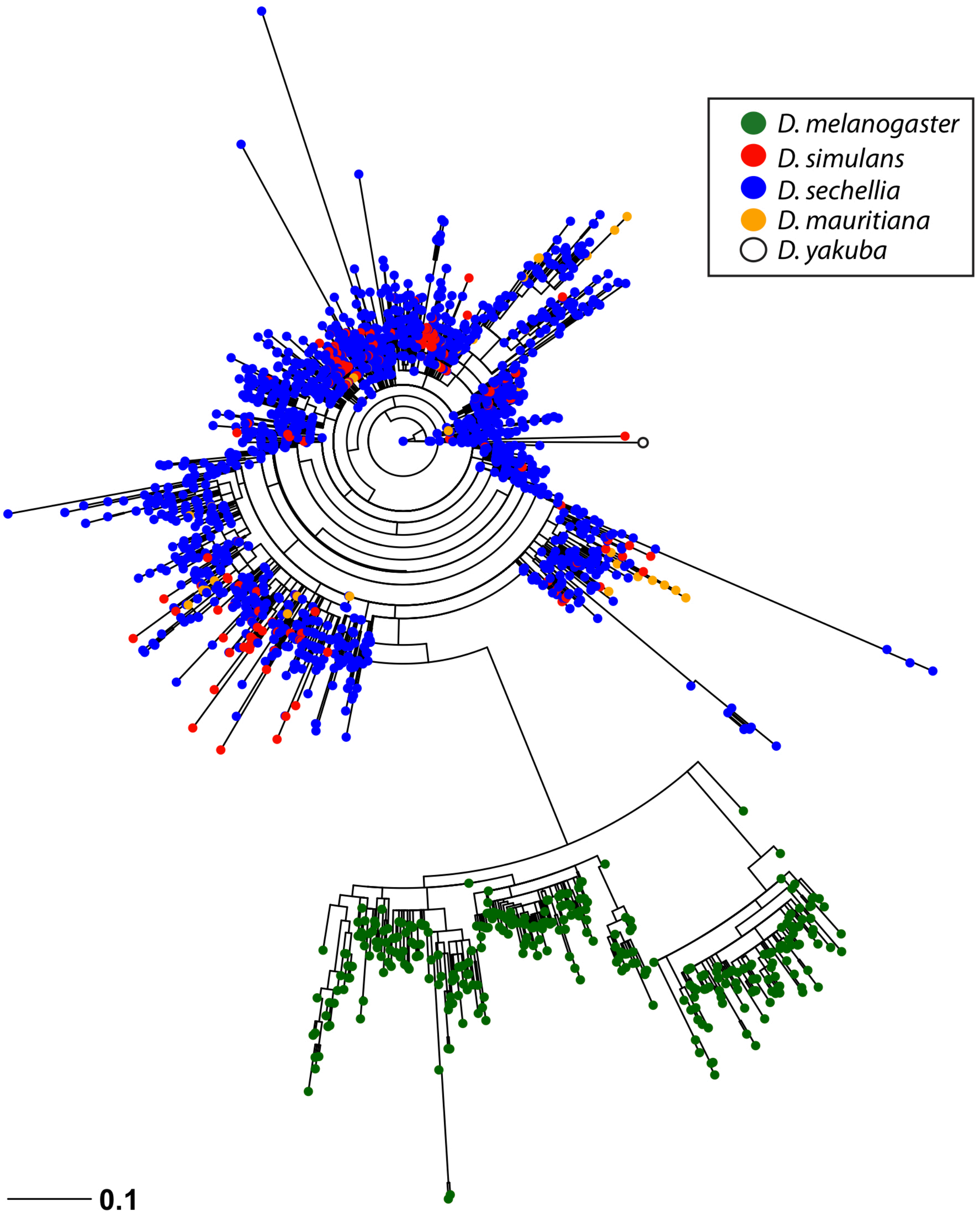

Figure S2. Maximum likelihood tree showing relationship between *Rsp* and *Rsp-like* repeats the *melanogaster* group.

Figure S3. Alignment of heterochromatic *Rsp* family repeats (canonical *Rsp* of *D. melanogaster* and *Rsp-like* of the *simulans* clade, *D. erecta* and *D. yakuba* repeats) and euchromatic *RlX* repeats in species of the *melanogaster* subgroup.

```
                                       1                                                50                                                100                                135
                                       |                                                |                                                 |                                  |
D. erecta Rsp-like-1                   TGCTACTGA-TTATGATCG-CCTGGTGGCAAAAATCGAGAAAGTCC-A-A-AAATATGCCATAAAAATGCG-CATTTTGT-A--GA-CAAAATT-TTAAAATTTTTCG-GTTTAAGTTACCAGGCGAACAGGA-ATTCC-
D. erecta Rsp-like-2                   TGCTACTGA-TCATGATCG-CCTGGTGCCAAAAATCGAACAAATTC-A-C-AAATTTGCCATAAAAATGCG-CATTTTGT-A--GG-CAAAATT-TTAAAATTTTTCG-GTTTATGTTAACAGACGAACAGGA-ATTCC-
D. yakuba Rsp-like                     ---------------------CTGGTGC-----AT--AA-A-A--C-AACGAAATGT-CC-CAAAAAC-CGG-ACTTT-TAAGCCG-AAAAATGG--AGA-TATTTCGAATTTCAGATACCAGGCAAACAGAA-------
D. mauritiana Rsp-like                 --CTACTG-ATTATCATCG-CCTGGTATCTAAAATCGAA-AGAT-CCA--AAAATTTTGCACAAAAATGCG-CATTTTGTAAGC---CA-AATTGAGAGA-TTTTTCG-GTTTAAACTACCAGGCGAACTG---------
D. sechellia Rsp-like                  --CTACTG-ATTATCATCG-CCTGGTATCTAAAATCGAA-AGAT-CCA--AAAATTTTGCACAAAAATGCG-CATTTTGTATGC---CA-AATTGAGAGA-TTTTTCG-GTTTAAATTACCAGGCGAA------------
D. simulans Rsp-like                   --CTACTG-ATTATCATCG-CCTGGTATCTAAAATCGAA-AGAT-CCA--AAAATTTTGCACAAAAATGCG-CATTTTGTAGGC---CA-AATTGAGAGA-TTTTTCG-GTTTAAACTACCAGGCGAACAG---------
D. melanogaster Rsp (Left canonical)   T-CTAGAG-ATTCTGTTCAAC-TGGTAAG-------CAA-AAA--C-A--GTAAA-TTGC-CTAAGTTTTA-CATTTT--AAGCGGTCA-AAATGGGTGA-TTTTCCG-ATTTCAAGTACCAGACAAACAGAAGATACCT
D. melanogaster Rsp (Right canonical)  T-CTACAG-ATTAT-TTAAACCTGGTA-CA------CAA-AAA--C-A--ATAAA-TTG-ACTAAGTTATGTCATTTT--AAGCGGTCA-AAATGGGTGA-TTTTTCG-ATTTCAAGTACCAGGCGAACAGAAGACACCT
D. melanogaster RlX-1                  --CTACTCAATTATGATC-GCCTAGTAACAAGCAACCAA-AAAT-CTAT-AAAATTTGCCACAAAAAACCA-TAATTTTTAGG--G-CA-AATTGTTTGA-TTTTTCG-ATTTAAATTACCAGGCGAACAG---------
D. sechellia RlX-1                     T--TACTGA-TTATGATC-ACTTGGTACCAAAAATCCAA-AAAC-CTAA-CAAATTCGGCACAAAATCGCG-CATTTTGTAAT--G-CA-AATTGTTTGA-TTTTTCG-GTTTAAATTACCAGGCGAACAG---------
D. simulans RlX-1                      T--TACTGA-TTATGATC-ACTTGGTACCAAAAATCCAA-AAAT-CTAT-CTAATTCGGCACAAAAATGCG-CATTTTGTAAT--G-CA-AATTGTTTGA-TTTTTCG-GTTTAAATTACCAGGCGAACAG---------
D. melanogaster RlX-2                  --CTACTCAATTATGATC-GCCTAGTAACAAGCAACCAA-AAAT-CTAT-AAAATTTGCCACAAAAAACC--TAATTTTTAGG--G-CA-AATTGTTTGA-TTTTTCG-ATTTAAATTACCAGGCGAACAG---------
D. sechellia RlX-2                     T--TACTGA-TTATGATC-ACTTGGTACCAAAAATCCAA-AAAC-CTAA-CAAATTCGGCACAAAATCGCG-CATTTTGTAAT--G-CA-AATTGTTTGA-TTTTTCG-GTTTAAATTACCAGGTGAACAG---------
D. simulans RlX-2                      T--TACTGA-TTATTATC-ACTTGGTACCAAAAATCCAA-AAAT-CTAT-CTAATTCGGCACAAAAATGCG-CATTTTGTAAT--G-CA-AATTGTTTGA-TTTTTCG-GTTTAAATTACCAGGCGAACAG---------
D. melanogaster RlX-3                  --CTACTCAATTATGGTT-GCCTAGTAACAAGTAACCAA-AAAT-CGAA-AAAATTAGCCACAAAAATCA--GCATTTTTAGG--G-CA-AATTGTTTGA-TTTTTCG-GCTTAAATTACCAGGCAAACAG---------
D. sechellia RlX-3                     T--TACTGA-TTATGATC-ACTTGGTACCAAAAATCCAA-AAAC-CTAA-CAAATTCGGCACAAAATCGCG-CATTTTGTAAT--G-CA-AATTGTTTGA-TTTTTTG-GTTTAAATTACCGGGTGAACAG---------
D. simulans RlX-3                      --CTACTCA-TTATGATC-ACTTGGTACCAAAAATCCAA-AAAT-CTAT-CTAATTCGGCACAAAAATGCG-CATTTTGTAAT--G-CA-AATTGTTTGA-TTTTTCG-GTTTAAATTACCAGGCGAACAG---------
```
